# Layer Adhesion Test of Additively Manufactured Pins: A Shear Test

**DOI:** 10.3390/polym14010055

**Published:** 2021-12-24

**Authors:** Márton Tamás Birosz, Mátyás Andó, Ferenc Safranyik

**Affiliations:** Savaria Institute of Technology, Eötvös Loránd University, 1053 Budapest, Hungary; am@inf.elte.hu (M.A.); sf@inf.elte.hu (F.S.)

**Keywords:** FDM, 3D printing, additive manufacturing, shear test, adhesion

## Abstract

Additive Manufacturing (AM) became a popular engineering solution not only for Rapid Prototyping (RP) as a part of product development but as an effective solution for producing complex geometries as fully functional components. Even the modern engineering tools, such as the different simulation software, have a shape optimization solution especially for parts created by AM. To extend the application of these methods in this work, the failure properties of the 3D-printed parts have been investigated via shear test measurements. The layer adhesion can be calculated based on the results, which can be used later for further numerical modeling. In conclusion, it can be stated that the layer formation and the structure of the infill have a great influence on the mechanical properties. The layers formed following the conventional zig-zag infill style show a random failure, and the layers created via extruded concentric circles show more predictable load resistance.

## 1. Introduction

Modern engineering practice involves many different tools to make the product design phase more effective. The implementation of Rapid Prototyping (RP) can drastically reduce the time of a full product design cycle [1]. This allows testing different ideas, stating conclusions, and making modifications without the risk of high tooling costs. As the technology evolved, several different RP technologies became available in the market. These allow working with different materials and building in different sizes from micro-scale engineering [2] to architectural scale (concrete printing) [3].

The parts made by these technologies are often complete end products, not just an intermediate phase of product development. The RP technologies can be divided into several groups regarding the raw materials or the forming procedures [4]. What they all have in common is that they build the desired geometry layer by layer in an additive way. Tooling geometry does not affect the shapes that can be formed, as at traditional subtractive processes. However, these layered structures have significant anisotropy.

Additive Manufacturing (AM) can be a charming way to create parts that are optimized for the smallest possible weight, deformation, or just generally to simulate its behavior [5,6,7]. Many Finite Element (FE) software offer a tool to perform Topology Optimization, create a Lattice Structure [8], or perform a Generative Design [9], and those can be manufactured most of the time only by 3D printing. However, these simulations are rarely considering the effect of the aforementioned anisotropy. The most widely used AM technology is Fused Deposition Modeling (FDM), which operates with thermoplastic raw material. The FDM technology is based on material extrusion and deposition. It melts the polymer and pushes it through a nozzle that has a standard diameter. The result is a continuous line with constant thickness. The process work by depositing layers upon layers, so it creates the 3D geometry. However, this layered structure results in significant anisotropy in the part [10,11], thus explicitly considering that print orientation is mandatory for each print. Numerous published works focus on the material properties and technological parameters. Zharylkassyn et al. [12] investigated the effect of layer thickness, printing temperature, and print orientation to find the optimal values for the best dimensional accuracy. Mohanty et al. [13] used the reputed Taguchi method for the same purpose. Pennington et al. [14] measured the ABS parts with a coordinate measurement machine and compared them to examine the effect of printing parameters on dimensional accuracy. Buj-Corral et al. [15] also investigated the dimensional errors and stated that with minimal layer thickness, high printing speed, and relatively low print temperature, the adverse effects can be minimalized. Torres et al. [16] performed a torsion shear test to create mechanical property optimization and found that the heat treatment can improve the performance of the parts. Dev and Srivastava [17] tried to find the parameters that optimized the flexural strength. The outcome of the mentioned research is the optimal geometry from the point of dimensional accuracy of the printed part. However, in reality, to find such an optimum for parts with complex geometries is a challenge; thus, we have to take the worst-case scenarios into account as well.

To extend the application of geometrical optimization, the main behaviors of AM need to be assessed. To create a proper material model for simulations, several material tests have to be conducted. One of the most important tasks is to measure the adhesive or cohesive bonding force between 3D-printed layers. The significant anisotropy of the parts created by FDM is well known, as the interlayer bonding is less resistant to load than the raw material. In this work, a shear test developed specifically for 3D-printed PLA parts is presented. This raw material is well examined in numerous published works [18,19,20], so most of the influencing factors of the printing can be considered.

In this paper to supplement the above-mentioned literature, the adhesion between the layers was examined. For this, several shear tests on cylinder samples have been performed with different layer formation methods to compare the adverse effects of the layered FDM parts.

## 2. Materials and Methods

For the experiments, Prusament PLA Extrafill Traffic White (Prusa Research, Prague, Czech Republic) was used. Table 1 contains the general properties of the PLA filament. This type of thermoplastic filament is one of the most frequently used raw materials for FDM printing due to its favoring printing behavior.

For creating the G-code, Prusa Slicer 2.3.3. slicing software, marketed by Prusa Research, Prague, Czech Republic, was used. The following parameters were used for 3D printing: layer thickness of 0.2 mm, nozzle diameter 0.4 mm, raster angle 45°, 100% infill, print speed 20 mm/s, extruder temperature 215 °C, bed temperature 60 °C, and extrusion multiplier 0.92. A Prusa i3 MK3S FDM 3D printer (Prusa Research, Prague, Czech Republic) was used to manufacture the samples.

**Figure 1:**
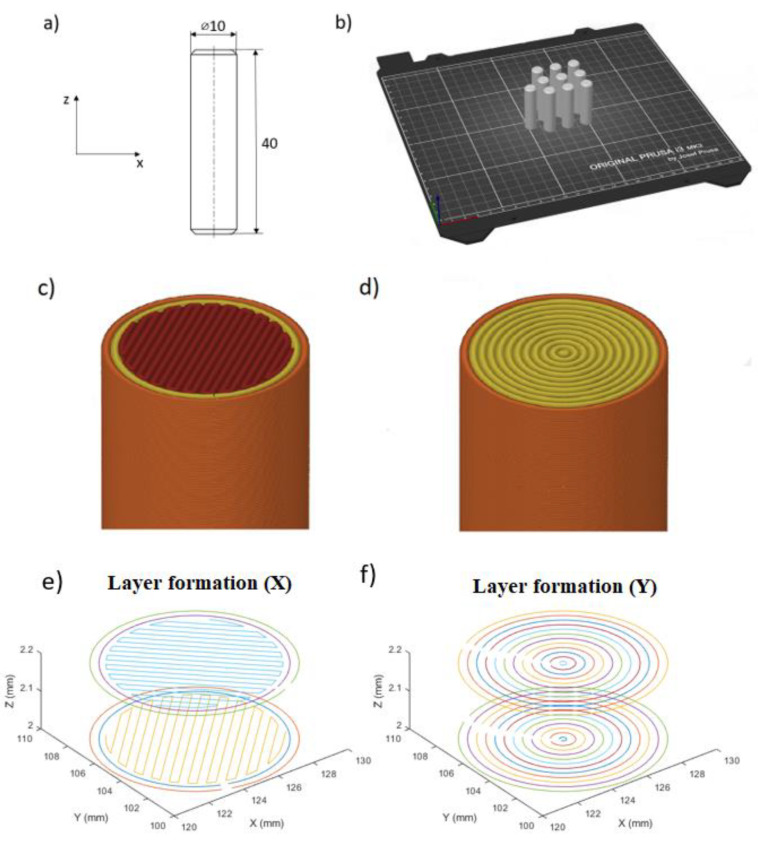
Test specimen: (**a**) geometry, (**b**) print orientation on the building plate, (**c**) layer structure of X specimens, (**d**) layer structure of O specimens, (**e**) layer formation of X specimens, and (**f**) layer formation of O specimens.

The geometry of the shear test specimen was a Ø10 mm and 40 mm long cylindrical sample (pin), as can be seen in Figure 1a. The geometry was chosen because the test is mostly related to the standard for fasteners according to [21]. The printing orientation for each piece was perpendicular to the build plate, as shown in Figure 1b. For the formation of the layers, two types of fillings were used. The first one uses two contour lines and fills the intermediate area with a continuous line drawn as a parallel zigzag path, and this filling is rotated 90° on each subsequent layer. This kind of infill is the default setup for most slicing software. The sample group of this layer form was marked with “X”. The second type uses only contour lines to create a solid sample. For the cylindrical specimens, this toolpath is an easy solution, but for more complex shapes, where the contour lines cannot be formed as concentric circles, the result will contain many air gaps inside the part. The sample group of these specimens was marked with “O”. The assumed difference between the two layer designs is based on the effect of previously experienced contour lines [20]. Therefore, the shear distribution is affected not only by the layering but also by the contour lines within the layers.

**Figure 2:**
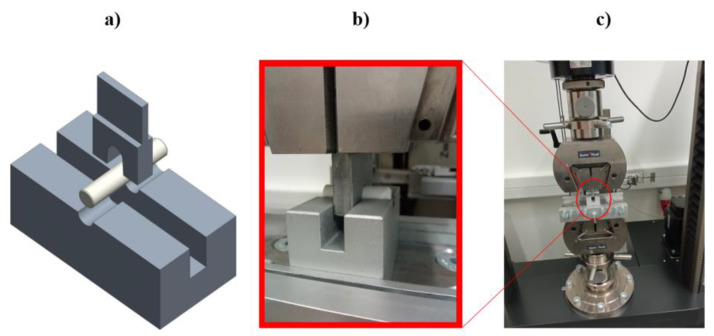
Test arrangement for the double shear test: (**a**) CAD model, (**b**) close view, (**c**) installed in the measuring machine.

The tests were conducted out on a Zwick Roell Z100 (obtained from Senselectro Ltd., Budapest, Hungary) universal material testing machine. Special fixtures were used, which were designed especially for this experiment [21] (Figure 2). The bottom part holds the specimens in place. It has a half-cylinder milled profile, which has the same nominal diameter as the specimens. The top part of the fixture (blade) has this same cylinder profile. By setting up a compression test environment on the universal testing machine, the middle part of the specimen is loaded by the blade into the groove of the bottom fixture. By the printing orientation, it was ensured that during the test, the load applied will always be perpendicular to the layers, so the interlayer adhesion connections were possible to examine with the transverse shear load. The test speed was 5 mm/min, and the force threshold was 80%. The test has been carried out at an air-conditioned room temperature of 22 °C with consolidated humidity. An optical microscope was used to inspect the loaded surfaces of the specimens, Zeiss Axio Imager A2.m (Carl Zeiss Microscopy, LLC, New York, NY, USA).

## 3. Results and Discussion

### 3.1. Explanation for the Test Results

**Figure 3:**
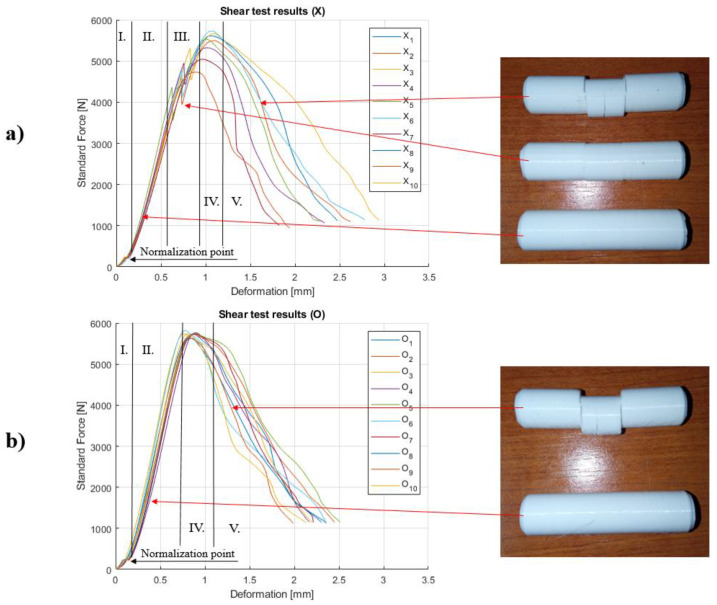
Shear test results with the main characteristic sections: (**a**) X samples and (**b**) O samples.

In Figure 3, the force–deformation curves are presented. The curves have special characteristics and the major zones are highlighted; these are denoted as I-V. At the beginning (I), as the load builds up the curves, both groups have a slightly less steep slope. Around 250 N load (≈0.11 mm deformation), this changes, and the curves straightened and then continue to rise steeply.

**Figure 4:**
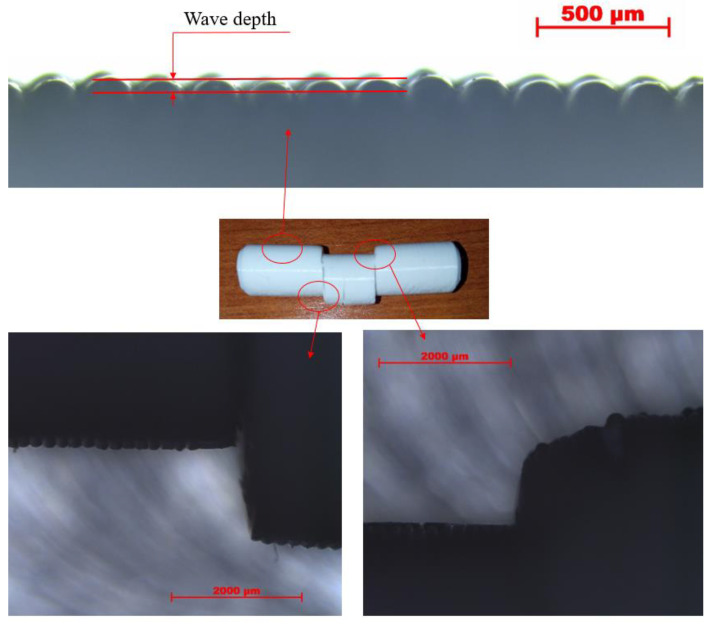
Representation of the flattening of the wavy surfaces.

The surface of the parts is wavy, due to the layered structure. At the initial phase of the transverse shear test, this roughness is settled on the tool surfaces. Figure 4 shows images of these surfaces taken with an optical microscope, at a 25 times magnification. The smoothing of the wavy by default surface on each face, which is connected with the tools, is present. There is a correlation between the wave depth of the surfaces and the normalization point of the measurement curves, beyond which purely the cross-sections are loaded and the characteristic of the curves changes. The wave depth on the boundary surface of the specimens is approximately 0.055 mm; thus, as loaded from two opposite directions, the deformation of these semi-cylindrical surface patterns correlates with the 0.11 mm deformation, where the normalization point was defined. Since this event occurs only below 5% of the overall measured force and deformation, it can be considered as an initial stage, which can be negligible.

After this initial stage, the slope of the curves gets straightened up (II). In the case of X, samples have an extra phenomenon made by stage III, where the force drops significantly when the cross-section cannot support more load and delamination occurs. The force drop is between 4000 and 5500 N. The O specimens did not show this behavior at all. Based on the interrupted shear test, a crack appeared in the middle of the samples (Figure 3). Based on the place of the crack, it is clear that it is created by the bending stress. It is also important that the crack and the crack propagation do not reduce the possible shear load capacity because the two types of the specimen have the same maximum measured force (stage IV). So, the infill formation tends to have an influence on the failure mode; thus, the effect of this parameter must be further investigated.

The difference of the layer filling method can be seen in Figure 1, where two joining layers can be seen also. As for the difference in layer formulation, there is no stage III in O samples. The contour lines of the X specimens lay on each other in every layer. The infill lines are also deposited on top of the previous layer, but in this case, the lines are crossed by each other.

**Figure 5:**
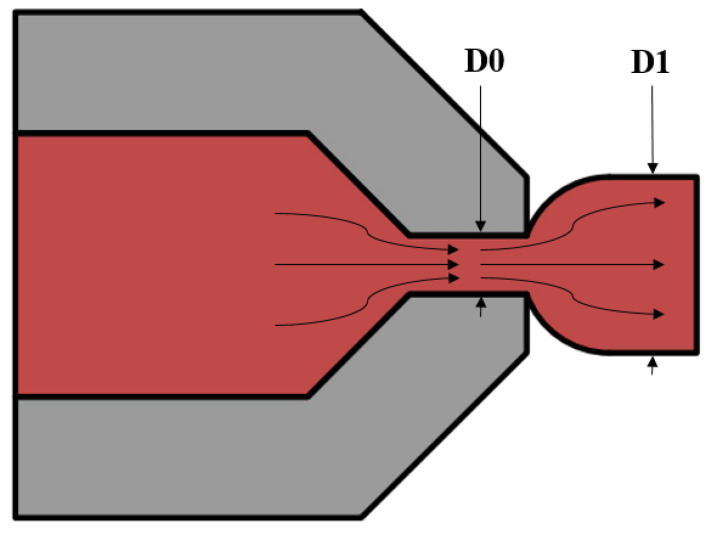
Schematic figure of the polymer expansion during extrusion.

Let us assume that each line that came out from the extruder nozzle of the 3D printer has a cylindrical cross-section. The nozzle has a 0.4 mm diameter hole, but based on the extrusion of the polymer [22], the actual diameter becomes slightly bigger (Figure 5). Since the layer height is 0.2 mm, the extruded lines will be compressed, and the cross-section will not be a perfect circle anymore; it will more likely be rectangular with fillets on the edges.

### 3.2. Difference between the Layer Filling Methods

**Figure 6:**
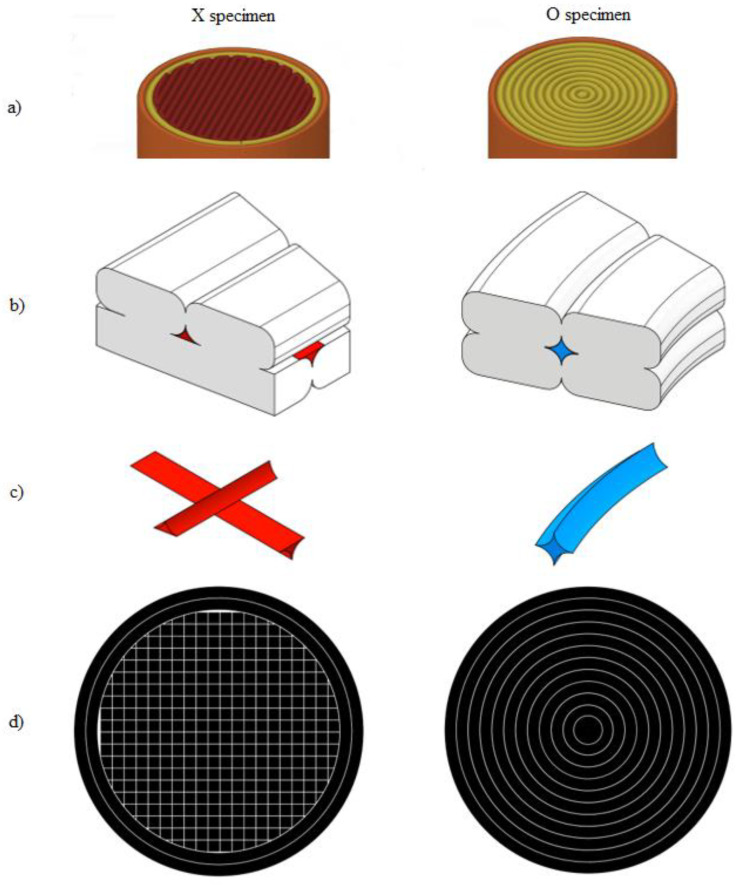
Effective bonded areas and ineffective areas between the lines of the layers: (**a**) layer structure, (**b**) lines extruded in the layers, (**c**) ineffective gap volumes, and (**d**) effective areas.

The sidewalls of the printed lines next to each other are connected, and an adhesive bond will form. This adhesive bond exists at the contact area above each specimen—in other words, between two layers. In the case of X specimens, the possible gaps are perpendicular to each other, but in the case of O samples, they are concentric (represented in Figure 6b in red and blue colors).

The slicer software is optimized to not let any air gap between the lines and layers of 100% infill be set. However, in reality, due to the cooling of the plastic and the unequal deposition force at the center and edge of the extruded lines, (resulting from the geometry of the nozzle), a gap is always presented. These gaps are pattern-dependent based on the printing set-up (Figure 6c,d). Between the lines of the specimens belonging to the O group, a four-pointed star shape represents the ineffective area. In the case of the X specimens, this gap only occurs in the case of the external contours (Figure 6a—brown color). However, in the infill region, the ineffective sections are rather V-shaped. The lines regularly cross each other so that the contact area is constantly interrupted, and it is divided into many smaller volumes. This phenomenon is ideal to crack propagation. That is the reason the X samples have force drop but the O samples do not.

However, because these mentioned gaps are smaller relatively to the whole specimen, the difference between the size of the functional bonding area is negligibly small, and only their location is significant in terms of load capacity. The results of the transverse shear tests confirm this statement, because the maximum force is the same in the two types of samples (stage IV).

In case of stage V, both of the specimens start to delaminate along the shear planes. After the shear stress becomes greater than the adhesion, then the samples start to delaminate. The shear curves do not show significant dropping along this stage.

## 4. Effect of the Contour Lines and Stress Distribution

Based on the results, there are two additional questions that we must further investigate:

1. How do the two contour lines affect the X samples?
2. Do the contour lines affect the maximum shear stress?

**Figure 7:**
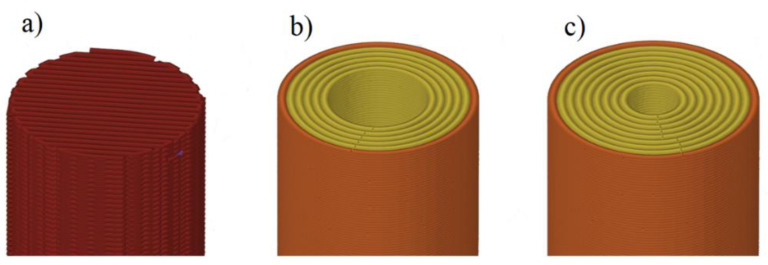
Checking layer formation, (**a**) 0 contour lines and 100% infill with X formation, (**b**) six contour lines and (**c**) nine contour lines.

Three sets of specimen groups have been prepared to examine these questions. One has zero contour lines and only 100% infill with the X zig-zag pattern (Figure 7a), and two other groups are of the O filling type, but using only six and nine concentric circles, leaving a hollowed middle section in the specimens.

**Figure 8:**
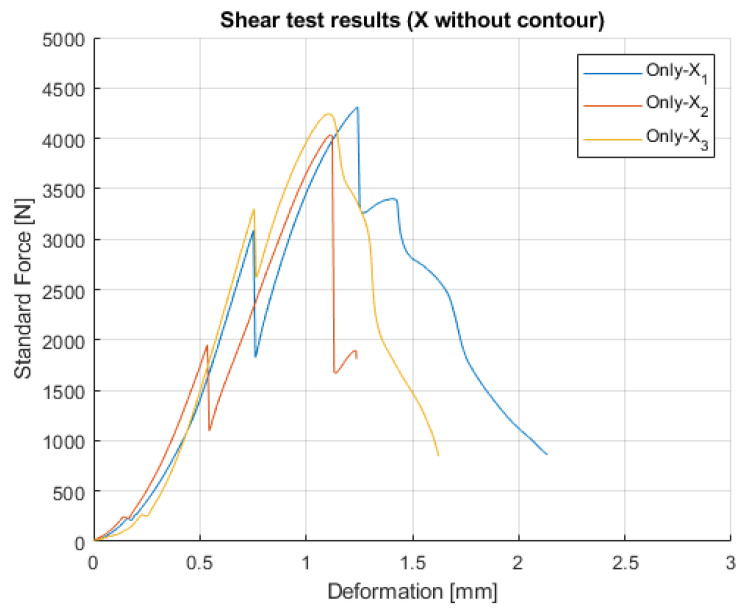
Results of X without contour lines specimens.

The results of the X specimens without contour lines are presented in Figure 8. The same force drop occurs but under a smaller load. Moreover, after the first force drop, a second and sometimes a third drop appear as well. In addition, there is breakage before the shear planes appear. This also proves that there is a vital role of the two contour lines related to the load resistance.

**Figure 9:**
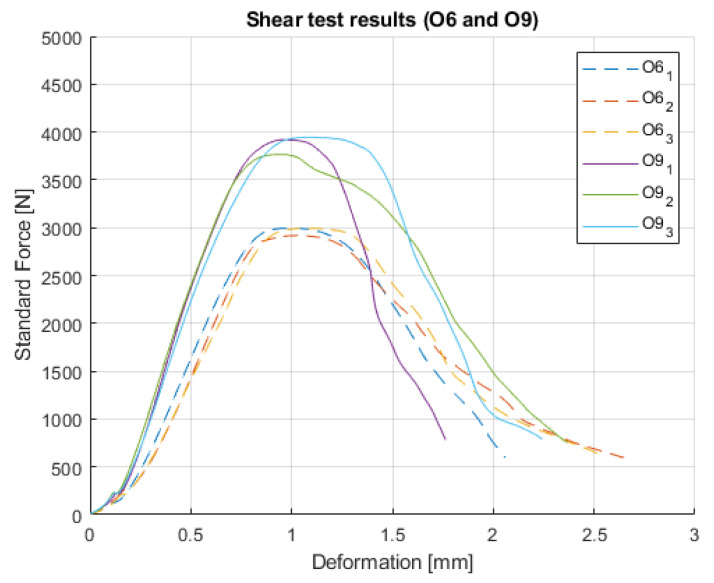
Results of O6 and O9.

The modified O samples act similarly to the original fully filled O specimens (Figure 9). The hollowed inner part in the pieces did not affect the shape of curves, only the load resistance.

The results of the three types of samples (O12-full, O9, and O6) help to calculate the shear stresses based on the following Equation (1) [23]:

(1) $$\tau \left(y\right)=\frac{V}{I}\cdot \frac{S\left(y\right)}{w\left(y\right)}.$$

*τ* is the shear stress value at a given point in the cross-section, *V* is the shear load in the specific cross-section, *S* is the 1st moment of area, *I* is the 2nd moment of area of the entire cross-sectional area (2), and *w* is the thickness in the material perpendicular to the shear. The equation for *I* is the following:

(2) $$I=\frac{\pi }{4}\left(r_{2}^{4}-r_{1}^{4}\right)$$

where *r_2_* is the outer diameter and *r_1_* is the inner diameter of the specimens (for O12, it is 0 mm).

**Figure 10:**
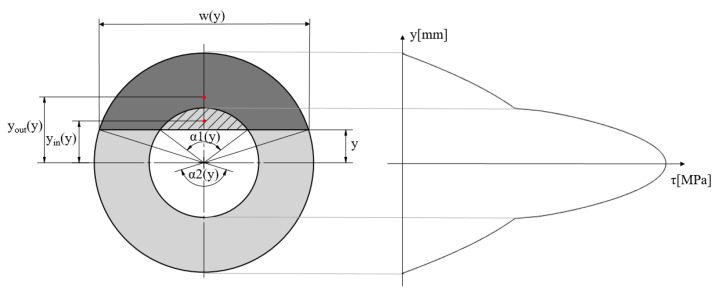
Geometry for shear stress calculation.

To calculate *S*, Equations (3)–(7) must be solved.

(3) $$S\left(y\right)=\left(A_{out}\left(y\right)\cdot y_{out}\left(y\right)\right)-\left(A_{in}\left(y\right)\cdot y_{in}\left(y\right)\right)$$

(4) $$A_{out}\left(y\right)=\frac{r_{2}^{2}}{2\left(\alpha_{2}\left(y\right)-\sin\left(\alpha_{2}\left(y\right)\right)\right)}$$

(5) $$A_{in}\left(y\right)=\frac{r_{1}^{2}}{2\left(\alpha_{1}\left(y\right)-\sin\left(\alpha_{1}\left(y\right)\right)\right)}$$

(6) $$y_{out}\left(y\right)=\frac{4r_{2}{\left(\sin\left(\frac{\alpha_{2}\left(y\right)}{2}\right)\right)}^{3}}{3\left(\alpha_{2}\left(y\right)-\sin\left(\alpha_{2}\left(y\right)\right)\right)}$$

(7) $$y_{in}\left(y\right)=\frac{4r_{1}{\left(\sin\left(\frac{\alpha_{1}\left(y\right)}{2}\right)\right)}^{3}}{3\left(\alpha_{1}\left(y\right)-\sin\left(\alpha_{1}\left(y\right)\right)\right)}$$

where *A_out_* is the area of the cross-section without the cut-out, *A_in_* is the area of the cut-out, *y_out_* is a centroid without the cut-out, *y_in_* is the centroid of the cut-out, and *α_1_* and *α_2_* are the central angles. Figure 10 contains an explanatory figure showing each marking.

**Figure 11:**
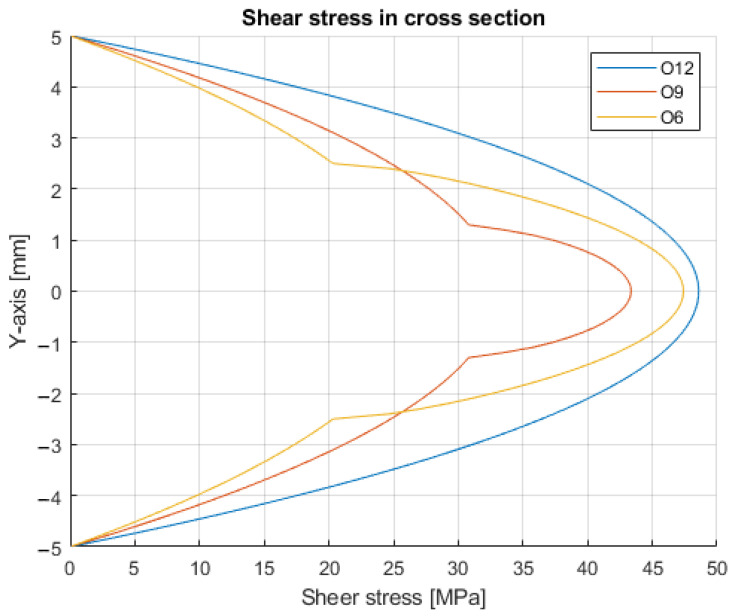
Shear stress distribution in the cross-sections.

By solving the equations for O12, O9, and O6, the maximal stress and the characteristic of its distribution in the cross-section can be seen (Figure 11).

Table 2 contains the numerical values for each tested group.

Based on the test results, the calculations show that the shear stress is approximately 46.1 MPa. The location of the maximum stress in all three cases is in the centroid of the cross-section.

Compared to other similar research works, this study shares some of the main findings; thus, the initial setup for the experiment was chosen, according to them, and the results can be compared. In [24], it is stated that the crack propagation for FDM parts will take place between the two layers if the load is applied perpendicular to the print orientation. In [25], it was found that the load resistance can be increased by reducing the layer thickness, and with an increased shear area, the value of shear force increase proportionally, as in the case of O samples in this study. In [16], the authors stated that the infill density should be maximized in order to maximize the strength and resistance to failure. Even though, in this paper, no hollowed specimens with a specific infill pattern were tested, this finding proved to be valid and can be extended. The difference between the X and O specimens is the division of the fully filled layer surface. Therefore, efforts should be made to keep the layers in contact with each other on a few large continuous (uninterrupted) surfaces as possible.

## 5. Conclusions

The special tools created for the transverse shear test can be properly used based on the consistently measured data. The fixation for both was sufficient to keep the gap between each other, and the experiment created the symmetrical failure of the specimens. From the results, the maximal shear strength can be predicted; these describe the adhesion properties under the applied conditions for PLA material. In conclusion, the following can be stated:

- The failure mechanism for the O specimens is well defined, and it follows a bilinear characteristic. However, the X specimens have a random initial failure mode, where some adhesive bonds break but the cross-section is still capable of holding more applied load.
- The predicted behavior can be ensured by adding more uninterrupted contact regions such as the tested concentric cylindrical infill structure.
- The obtained result is that for O-type layer formation, the shear stress would be around 46.1 MPa, and this value can later be used for failure prediction and numerical simulation.

The results can be used for creating an appropriate material model for Finite Element Analysis. For maximum load resistance, choose the O infill pattern. This structure is not always feasible due to the complexity of the 3D printed geometry, but the results of this paper show that to some extent, even hollow pieces have greater shear resistance and predictable failure.

## Figures and Tables

**Table 1:** PLA material properties [20].

Material	PLA
Filament diameter [mm]	1.75
Printing temperature [°C]	190–220
Bed temperature [°C]	50–60
Density [g/cm^3^]	1.24
Color	White
Tensile strength [MPa]	~47
Tensile modulus [MPa]	2100

**Table 2:** Numerical results of the test.

	Force (N)		Shear Stress (MPa)
	Mean	Std. Dev.	
O12	2862	49.9	47.6
O9	1939	39.9	43.4
O6	1496	29.4	47.4

## Data Availability

The data that support the findings of this study are available from the corresponding author, M.B., upon reasonable request.
